# Nicking Endonuclease-Mediated Vector Construction Strategies for Plant Gene Functional Research

**DOI:** 10.3390/plants9091090

**Published:** 2020-08-25

**Authors:** Qi Gong, Bin Wang, Xubiao Lu, Jiantao Tan, Yuke Hou, Taoli Liu, Yao-Guang Liu, Qinlong Zhu

**Affiliations:** 1 State Key Laboratory for Conservation and Utilization of Subtropical Agro-Bioresources, Guangzhou 510642, China; gongqi@stu.scau.edu.cn (Q.G.); wbin313@gmail.com (B.W.); TJT@scau.edu.cn (J.T.); houyuke@stu.scau.edu.cn (Y.H.); liutaoli@stu.scau.edu.cn (T.L.); 2 Guangdong Laboratory for Lingnan Modern Agriculture, Guangzhou 510642, China; 3 College of Life Sciences, South China Agricultural University, Guangzhou 510642, China; lxbiao648@gmail.com

**Keywords:** nicking endonuclease, DNA fragment assembly, NEMDA, vector construction, RNAi, CRISPR/Cas9

## Abstract

Plant genetic engineering vectors, such as RNA interference (RNAi) and CRISPR/Cas9 vectors, are important tools for plant functional genomics. Efficient construction of these functional vectors can facilitate the study of gene function. Although some methods for vector construction have been reported, their operations are still complicated and costly. Here, we describe a simpler and low-cost vector construction method by nicking endonucleases-mediated DNA assembly (NEMDA), which uses nicking endonucleases to generate single-strand overhanging complementary ends for rapid assembly of DNA fragments into plasmids. Using this approach, we rapidly completed the construction of four RNAi vectors and a CRISPR/Cas9 knockout vector with five single-guide RNA (sgRNA)-expression cassettes for multiplex genome editing, and successfully achieved the goal of decreasing the expression of the target genes and knocking out the target genes at the same time in rice. These results indicate the great potential of NEMDA in assembling DNA fragments and constructing plasmids for molecular biology and functional genomics.

## 1. Introduction

With the release of more and more various plant genome data, the study of gene functions has become increasingly important. As an important tool for plant gene function research, genetic engineering vectors have affected the progress of plant functional genomics [1]. Among genetic engineering vectors, RNA interference (RNAi) vectors and the CRISPR/Cas editing system are currently the most commonly-used reverse genetic tools for studying gene functions [2].

Construction of RNAi vectors, having an intron-containing hairpin RNA (ihpRNA) structure, and CRISPR/Cas9 vectors, for multiplex genome editing vectors, actually involves the splicing of multiple DNA fragments. Therefore, an efficient method for linking or assembling multiple DNA fragments is very important to improve the efficiency of vector construction. A number of technologies for multi-fragment assembly have been developed, such as overlap extension-PCR (OE-PCR) [3,4], BioBrick [5,6], Gateway recombination [7], sequence and ligation-independent cloning (SLIC) [8], Golden Gate cloning [9,10] and Gibson Assembly [11]. Among them, Golden Gate cloning and Gibson Assembly are the most commonly-used methods. Golden Gate cloning is a “seamless” cloning strategy mediated by type IIS restriction endonuclease. Type IIS restriction endonuclease, such as *Bsa*I, cuts the DNA outside the recognition site to produce DNA fragments with single-strand ends, and then the DNA fragments with supplementary single-strand ends are sequentially inserted into the target construction by DNA ligase. However, since the complementary single-strand end is only 4 bp, the connection efficiency of this method is greatly reduced, which is a major limiting factor in the application of this method [12]. The Gibson Assembly produces single-strand overhanging ends through T5 exonuclease, and then DNA fragments are linked by the actions of *Taq* DNA polymerase and *Taq* DNA ligase [11]. T5 exonuclease is highly active and may completely hydrolyze strands of short fragments, so this method is not suitable for the assembly of small fragments. In addition, the price of the Gibson Assembly kit is relatively expensive, which limits the widespread use of this method [13]. Therefore, it is desirable to develop an efficient and inexpensive multi-fragment assembly method.

Nicking endonucleases (NE) is a group of enzymes that recognize specific DNA sequences in double-stranded DNA and introduces nick only in one of the strands [14]. NEs hydrolyzing the top strand in a duplex (5’→3’/3’→5’) is denoted as Nt, and the one acting upon the bottom strand, Nb. NEs are widely used in isothermal amplification, gene mapping, sparing DNA cloning that requires no enzymatic ligation, as well as the basis for construction of chimeric proteins of predetermined specificity [15]. In addition, through NE nicking single strand to produce single-strand complementary overhanging ends, NEs have been applied to T-A cloning of DNA fragments [16].

In this study, according to the characteristics of NEs, we developed a simple and efficient nicking endonucleases-mediated DNA assembly (NEMDA) method. By this method, we rapidly constructed plant ihpRMAi vectors and assembled multiple sgRNA expression cassettes into a CRISPR/Cas9 vector for multiplex genome editing, and we confirmed the reliability and functionality of these constructs by transient expression in tobacco leaves and stable transformation of rice. Therefore, this study provides a new, simple, and low-cost vector construction method for DNA fragments assembly in molecular biology research.

## 2. Results and Discussion

### 2.1. Development of Nicking Endonuclease-Mediated DNA Assembly Strategy for Constructing ihpRNA Vectors

**Figure 1:**
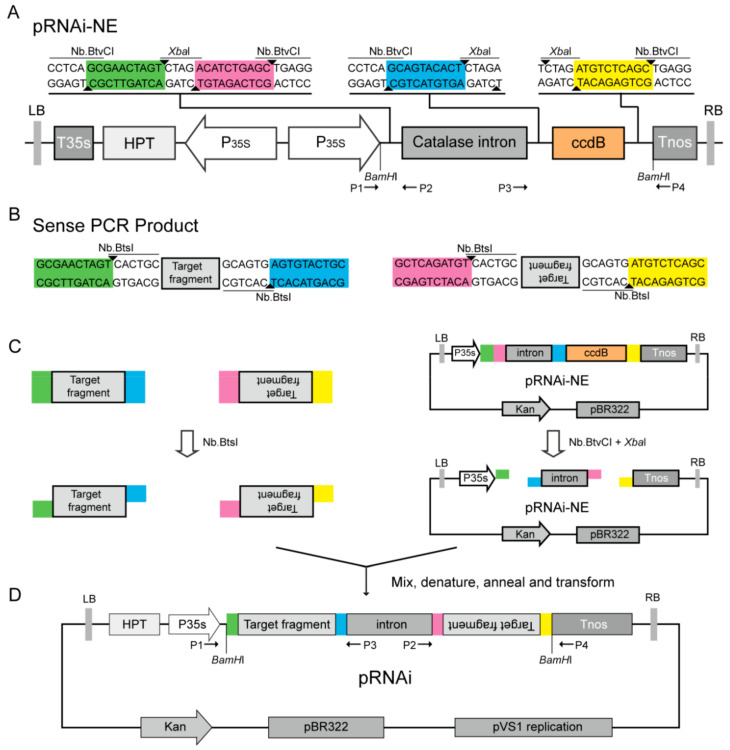
Schematic diagram of the nicking endonucleases-mediated DNA assembly (NEMDA) strategy for plant ihpRNA vector constructions. (**A**) The pRNAi-NE includes the 35S CaMV promoter, the Catalase intron, the *ccdB* gene, and four Nb.BtvCI and *Xba*I recognition sites with differently designed adaptors (different colors). (**B**) The sense and antisense PCR products have four Nb.BtsI recognition sites with differently designed adaptors (different colors). (**C**) One-step construction of an ihpRNA vector. The target fragments of the gene of interest are PCR amplified using gene-specific primers carrying Nb.BtsI sites and adaptors complementary to the appropriate sequences on the vector. The unpurified PCR products digested by Nb.BtsI are mixed, in one tube, with unpurified pRNAi-NE vector digested by Nb.BtvCI and *Xba*I, for heat-inactivation of these restriction endonucleases and melting out of the nicked end strands, annealing. The T4 DNA ligase also can be used to increase cloning efficiency. The reaction product is transferred into *E. coli* competent cells to produce the pRNAi plasmid (**D**).

In order to use the NEMDA strategy for fast and high-throughput assembly of ihpRNA, we developed a plant basic RNAi vector pRNAi-NE (Figure 1A), which was obtained by assembling a NE-RNAi fragment (~1.2 kb) into our previous plant binary vector pYLRNAi.2.0 [17] using modified Gibson cloning [13] (Supplementary Figure S1A). The functional element of pRNAi-NE is composed of *CaMV35s* promoter, first multiple cloning site (MCS1), the *GUS* catalase intron, MCS2 and *Nos* terminator (Figure 1A), flanked by *BamH*I sites for digestion and identification of the ihpRNA construct. MCS1 contains two Nb.BtvCI and one *Xba*I sites, and MCS2 contains a *ccdB* gene as a negative screening marker, with one Nb.BtvCI site and one *Xba*I site in both flanking sides of *ccdB*. Both ends of the sense and antisense amplification primers of the target fragment contain an Nb.BstI site, which generate single strand complementary overhanging ends to the vector and intron fragment, respectively. After nicking and cutting pRNAi-NE with Nb.BtvCI and *Xba*I, and nicking the sense and antisense fragments with Nb.BtsI, these unpurified reaction products are mixed, treated at 80 °C for 20 min (to melt out the nicked short strands), and annealed at room temperature. Finally, this mixture is used to directly transformed *Escherichia coli* competent cells, such as DH5α and DH10B. Only recombinants (without *ccdB* gene) with the correct assembly of sense, intron, and antisense fragments were grown. The recombinant pRNAi-NE binary vectors are used for *Agrobacterium* for plant transformation.

Compared with other methods, the NEMDA strategy for assembly of multiple fragments in vector construction has the following advantages: (1) NEMDA requires less time to assemble DNA fragments and does not require fragment purification and option use or no use of T4 DNA ligase (use of T4 DNA ligase may increase the cloning efficiency [18]); (2) pRNAi-NE contains the *ccdB* expression cassette, which produces zero background for ihpRNA construct; (3) the single-strand end produced by this method is much longer than the IIS restriction endonuclease, thus the cloning efficiency can be higher.

### 2.2. The NEMDA-Mediated Rapid Splicing of Plant ihpRNA Constructs With pRNAi-NE

In order to test the construction efficiency of ihpRNA constructs based on the pRNAi-NE vector, we amplified the target sequences of four genes *GFP*, *GUS*, *NbPDS,* and *OsPDS*, which were digested with Nb.BstI, and then mixed the pRNAi-NE plasmid digested with Nb.BtvCI and *Xba*I. After the process of denaturation and annealing, the mixture was transferred into *E. coli* DH10B cells, and the transformants were selected on the LB (Luria-Bertani) plate containing kanamycin. The sense primers of each gene were used for PCR detection of recombinants. Colony PCR results of randomly selecting 12 clones for each vector showed expected bands in agarose gel (Supplementary Figure S2A–D). For each construct, one clone plasmid was chosen for *BamH*I digestion analysis. As shown in Supplementary Figure S2E, all constructs contain the correct insert, indicating the high cloning efficiency of the ihpRNA constructs.

### 2.3. Silencing Marker Genes in Tobacco Leaves and Endogenous OsPDS in Rice by Assembling ihpRNA Constructs

**Figure 2:**
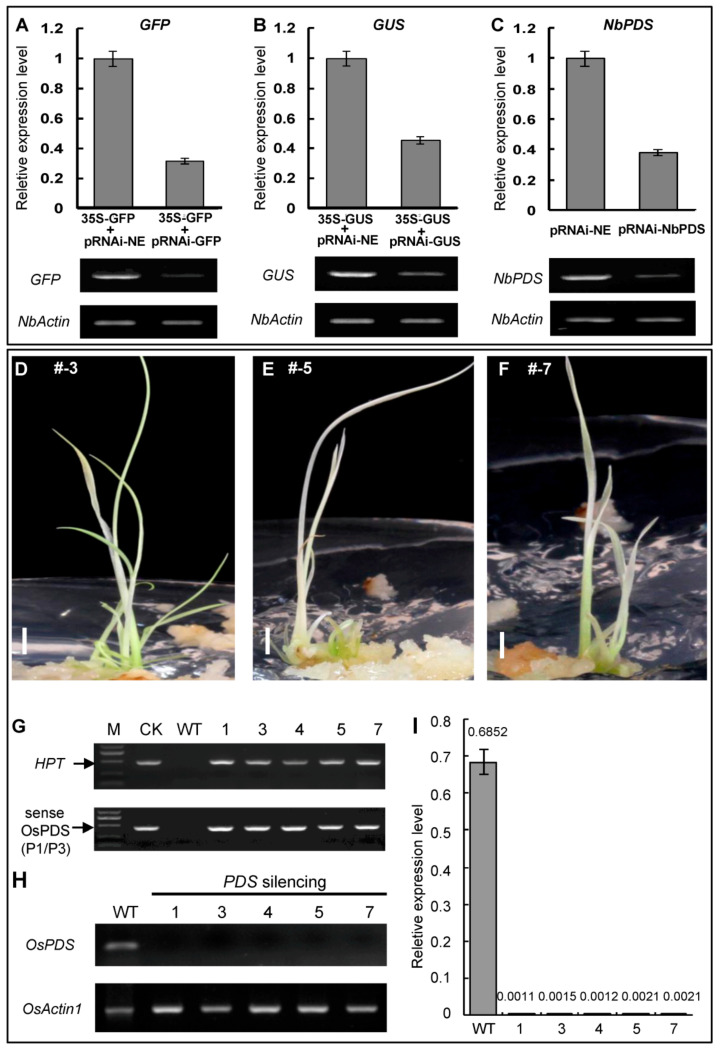
Functional detections of plant ihpRNA constructs by the silencing of two marker genes (*GUS* and *GFP*) and two endogenous genes (*NbPDS* and *OsPDS*). (**A**–**C**) qRT-PCR and RT-PCR were performed to analyze the silencing effect of *GUS*, *GFP,* and *NbPDS* genes in tobacco leaves. *NbActin* was used as internal control. The results were analyzed using the formula of the comparative Ct method (2^−ΔΔCt^). (**D**–**F**) Typical albino leaves phenotype of transgenic rice lines caused by *OsPDS* gene interference with pRNAi-OsPDS. Scale bars represent 5 mm. (**G**) *Hpt* gene and *OsPDS* sense fragments were detected in transgenic lines. (**H**–**I**) RT-PCR and qRT-PCR analyses were performed to assess the silencing effect of *OsPDS* genes. *OsActin1* was used as internal control. These results were calculated as differences in the cycle threshold (Ct) between *OsPDS* and *OsActin1* (2^−ΔCt^). Error bars represent standard deviations (SD) of three independent experiments.

Co-agroinfiltration has been widely used to transiently express genes or verify the effect of RNAi constructs for marker genes silencing [19,20,21]. In order to demonstrate that if the above ihpRNA constructs can be used for gene silencing, we first tested the effect of pRNAi-GFP and pRNAi-GUS through agroinfiltration transient expression in tobacco leaf. The *Agrobacterium* containing pCMBIA1302 (35S: GFP) or pCMBIA1305 (35S:GUS) is mixed with the *Agrobacterium* containing pRNAi-GFP or pRNAi-NE (control) and pRNAi-GUS or pRNAi-NE (control), respectively. The mixed culture of *Agrobacterium* cells was co-infiltrated into different parts of the same leaf of tobacco. The semi-quantitative RT-PCR results of the co-agroinfiltrated leaf regions showed a decrease in the transcription level of these marker genes, which were further confirmed by quantitative RT-PCR (qRT-PCR) analysis, showing that the expression levels of *GFP* and *GUS* were reduced to ~40% and ~30% of the control, respectively (Figure 2A,B). The *PDS* (phytoene desaturase) gene is the most commonly used marker gene for studying gene silencing in plants [19]. Thus, we also tested the transient silencing of the tobacco endogenous *NbPDS* gene, using the pRNAi-NbPDS construct. After agroinfiltration of pRNAi-NbPDS, the RT-PCR results indicated that the mRNA level of *NbPDS* was significantly reduced in tobacco leaves, and the qRT-PCR analysis showed that the expression level of *NbPDS* in the treated tobacco leaf was reduced to 38% of the control (Figure 2C). These results indicated that the ihpRNA constructs in this study are efficient and functional.

To further confirm that the assembled ihpRNA vectors can be used for stable endogenous gene silencing in plants, we transferred pRNAi-OsPDS into *japonica* rice ZH11 through the *Agrobacterium*-mediated method. By detecting the *Hpt* and *OsPDS* fragments of genomic DNA, the plants containing the transgenic construct were screened (Figure 2G). The transgenic lines showed typical albino phenotype (Figure 2D–F). The sRT-PCR and qRT-PCR analyses showed that the expression of *OsPDS* in transgenic lines were greatly reduced (Figure 2H,I). These results showed that the ihpRNA constructs could steadily decrease the mRNA levels of target endogenous genes. The above results indicate that the NEMDA method can efficiently construct functional ihpRNA vectors, implying that this one-step assembled strategy has broad application prospects.

### 2.4. Fast Assembly of Multiple sgRNA Expression Cassettes in CRISPR/Cas9-Vector for Multiplex Genome Editing

**Figure 3:**
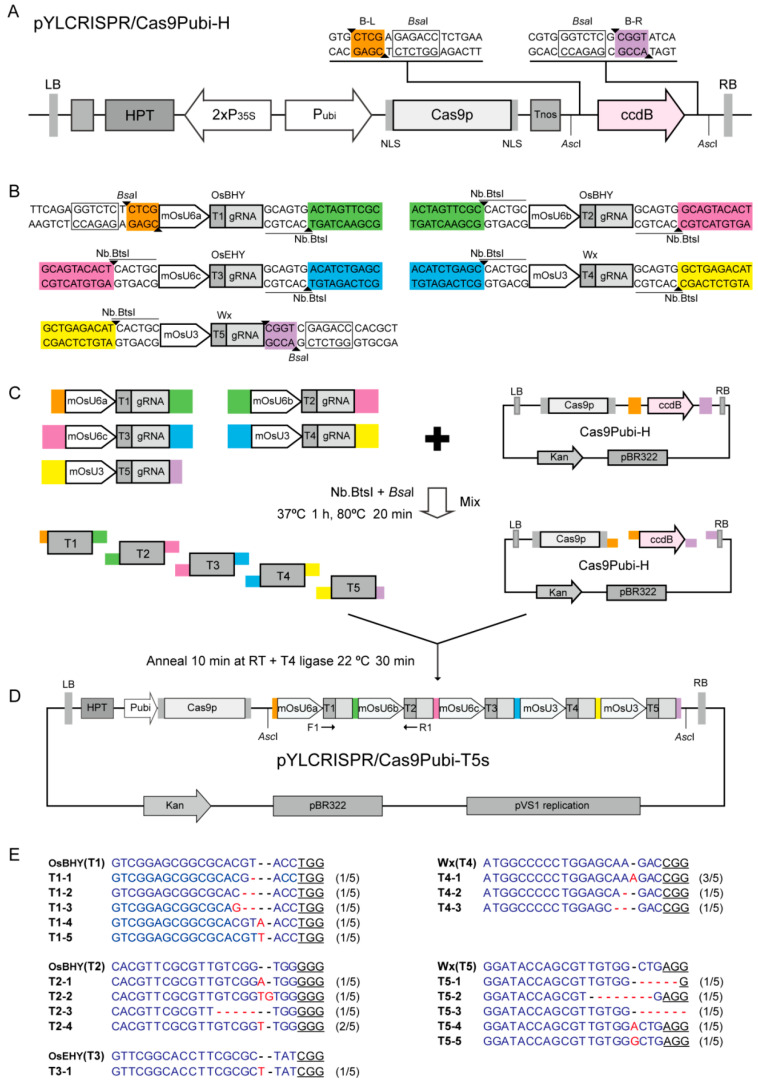
Schematic diagram of NEMDA strategy for plant CRISPR/Cas9 multiplex CRISPR/Cas9 vector construction and its mutation detection of transgenic plants. (**A**) Structural features of pYLCRISPR/Cas9Pubi-H with two *Bsa*I sites in the flanking sides of a lethal *ccdB* gene [22]. (**B**) The five sgRNA expression cassettes have *Bsa*I and Nb.BtsI recognition sites with differently designed target sequence adaptors (different colors). (**C**) One-step construction of multiple sgRNA expression cassettes into the CRISPR/Cas9 vector. Each sgRNA expression cassette is PCR amplified using primers carrying *Bas*I and Nb.BtsI sites and 10-bp complementary overhanging sequences. These PCR products were mixed with pYLCRISPR/Cas9Pubi-H vector, and treated with Nb.BtvCI and *Bsa*I, in one tube, for digestion, heating, annealing, and ligation. The reaction product was transferred into *E. coli* competent cells to produce pYLCRISPR/Cas9Pubi-T5s (**D**). (**E**) Results of multiplex genome editing for three rice genes (five targets) using the above plasmid. Each target site was effectively edited by randomly selecting five T-A clones for sequencing. Nucleotides variations of insertion or deletion present as red in targets. The PAM (protospacer adjacent motif) sequences of the target site are underlined. The number in brackets indicates the proportion of variants.

CRISPR/Cas9-mediated multiplex genome editing requires assembly of multiple sgRNA expression cassettes in single vector [22]. In our previous studies, we developed a high-efficiency plant CRISPR/Cas9 multiplex genome editing system by Golden Gate method (i.e., using *Bsa*I digestion) to link multiple sgRNA expression cassettes [22,23]. To verify the NEMDA method in the vector construction, we selected three rice genes (*OsBHY*, *OsEHY* and *OsWaxy*) to design five target sites (T1–T5), and used them to prepare a construct for multiplex editing based on our binary vector pYLCRISPR/Cas9pubi-H (Figure 3A). According to our previous protocol [22], to introduce the target sequences, the chimeric primers containing the target sequence were first used to amplify the shortened small nuclear RNA (snRNA) promoters [24] and sgRNA scaffold sequence, respectively. And then the integrated sgRNA expression cassettes were amplified by overlapping PCR using nick endonuclease site-containing specific primers (Supplementary Table S2), which generated 10-bp end complementary sequences for NEMDA. After overlapping PCR, we obtained five sgRNA expression cassettes with Nb.BtsI-cutting sites (Supplementary Figure S3A); among them the 5′ end and 3′ end of T1- and T5-sgRNA expression cassettes retained the *Bsa*I site for ligation into pYLCRISPR/Cas9pubi-H (Figure 3B). The purified PCR products and the pYLCRISPR/Cas9 plasmid were mixed and digested with Nb.BtsI and *Bsa*I, heated at 80 °C for 20 min, annealed at room temperature, and then ligated with T4 DNA ligase for 30 min. Finally, the mixture was transferred into *E. coli* competent cells (Figure 3C). Colony PCR analysis (using primers F1/R1) showed that all 12 randomly selected clones had all the fragments to be linked into the vector (Supplementary Figure S3B). Through restriction enzyme-digestion and sequencing, we successfully obtained the Cas9Pubi-T5s vector containing the complete 5 sgRNA expression cassettes as designed (Figure 3D and Supplementary Figure S3C).

Through *Agrobacterium*-mediated rice calli transformation, we further analyzed the function of the Cas9Pubi-T5s construct. After PCR amplifying each target site from DNA of transformed rice calli for T-A cloning, and then randomly selecting five positive clones from each target for sequencing, the results show that the above five targets were successfully edited, and produced different mutations in rice calli (Figure 3E). Except for the low editing efficiency using the shortened snRNA promoter mOsU6c as reported previously [24], the frequencies of the remaining mutations were high: almost 100% (5/5) (Figure 3E).

These results demonstrate that the NEMDA method can effectively achieve assembly of multiple DNA fragments (e.g., sgRNA expression cassettes) in a designed order in a single reaction. Compared with Golden Gate cloning, that uses the type IIs restriction endonucleases to produce short different overhanging ends [12], use of nicking endonucleases can flexibly produce longer single-strand overhanging complementary ends, which is more conducive to the assembly of multiple DNA fragments in vector construction. Therefore, this NEMDA method has great potential for studies of molecular biology and functional genomics.

## 3. Materials and Methods

### 3.1. Plant and Plasmid Materials

Tobacco (*Nicotiana benthamiana* Domin) was grown in a growth chamber under standard conditions at 25 °C under 16-h-light and 8-h-dark cycle and was used for genes transient silencing analysis. Rice (*Oryza sativa* L. vs. ZH11) was utilized for genes silence analysis of in vivo stable transformation. Plasmids pCAMBIA1305 and pCAMBIA1302 were used to the transient expression of GUS and GFP, respectively. The binary vector pYLRNAi 2.0 [17] and pYLCRISPR/Cas9pubi-H [22] were constructed by our previous studies. *E. coli* strains DH10B and Top10F’ (in which the ccdB gene is not lethal), and *Agrobacterium tumefaciens* EHA105 and GV3101 are preserved in our laboratory. YEP medium (Bacto-Trypton, 10 g/L; yeast extract, 10 g/L; NaCl, 5 g/L; pH 7.0) was used to culture *Agrobacterium*. The infiltration buffer (50 mM MES pH 5.6, 10 mM MgCl_2_, and 100 mM acetosyringone) was used to assist *Agrobacterium* infection.

### 3.2. Plant RNAi Basic Plasmid pRNAi-NE Construction

The binary plasmid pRNAi-NE was produced using our previous pYLRNAi 2.0 [17] as the skeleton and a splicing NE-RNAi fragment for replacing multiple coding sites (MCS) of pYLRNAi 2.0 (Supplementary Figure S1A). To obtain the pRNAi-NE, we first amplified the catalase intron fragment from pCAMBIA1305 with primers Fi-1/Ri and Fi-2/Ri, and *ccdB* fragment from pYLRNAi 2.0 with primers Fc/Rc-1 and Fc/Rc-2, through two rounds of PCR, respectively. Then, the two fragments were directly spliced by overlapping PCR to generate about 1.2-kb NE-RNAi fragment, which has the 30- and 25-bp overlapping sequences at both ends the same as the sequences on both sides of the insertion position of pYLRNAi 2.0 (Supplementary Figure S1B). Finally, the NE-RNAi fragment was inserted into pYLRNAi 2.0 by modified Gibson cloning method [13], using pYLRNAi 2.0 as a template and the NE-RNAi as a megaprimer. The recombinant pRNAi-NE was identified by colony PCR and *BamH*I-digestion (Supplementary Figure S1C,D). pRNAi-NE was maintained in *E. coli* TopF’ and used to generate all of the ihpRNA constructs to silence the genes used in this study. All used primers are listed in Supplementary Table S1.

### 3.3. Nicking Endonuclease-Mediated Plant ihpRNA Vector Constructions Using pRNAi-NE

Only sense and antisense PCR fragments are required to construct the ihpRNA vectors for silencing the gene of interest with pRNAi-NE by NEMDA, using the universal primers pair Fs/Rs (Fs, 5′-GCGAACTAGTCACTGC-gene specific forward sequence-3′ and Rs, 5′-GCAGTACACTCA CTGC-gene specific reverse sequence-3′) and Fa/Ra (Fa, 5′-GCTCAGATGTCACTGC-gene specific reverse sequence -3′ and Ra, 5′-GCTGAGACATCACTGC-gene specific forward sequence -3′) with nicking endonuclease sites to amplify the target gene to prepare the sense and antisense fragments, respectively. Purified PCR fragments (each ~500 ng) were digested by five units Nb.BtsI (NEB) at 37 °C for 1 h in a total volume 20 μL. pRNAi-NE was digested with NbBtvCI (NEB) and *Xba*I (NEB) at 37 °C for 1 h following 20 min of heat inactivation at 80 °C. Then, mixtures of unpurified sense and antisense digested-fragments (each ~50 ng) and digested pRNAi-NE plasmid (~100 ng) were incubated at 80 °C for 20 min for denaturation (producing single-strand overhanging complementary ends), followed by renaturation at room temperature. Finally, the mixture was precisely transformed into *E. coli* competent cells. In this way, all tested ihpRNA constructs for silencing *GUS*, *GFP*, *NbPDS,* and *OsPDS* genes were produced by NEMDA and named as pRNAi-GUS, pRNAi-GFP, pRNAi-NbPDS, and pRNAi-OsPDS, respectively. These plasmids were confirmed by PCR, digestion, and DNA sequencing. Primers used in ihpRNA constructs are listed in Supplementary Table S1.

### 3.4. Nicking Endonuclease-Mediated Multiple sgRNA Expression Cassettles Assembly for CRISPR/Cas9 Multiplex Genome Editing

Each sgRNA expression cassette is composed of three parts, contain a snRNA promoter, target sequence, and gRNA. Thus, according to our previous method [22], we used overlapping PCR to introduce target sequence into each sgRNA expression cassette. Briefly, the first round of PCR (20 μL) used four primers, the universal U-F and gRNA-R (0.2 mM each), and two target sequence-containing chimeric primers OsU#T#-R and gRT#-F (0.1 mM each), 0.2 U of high-fidelity DNA polymerase KOD FX, and pYLgRNA-mOsU# plasmids [24] (~20 ng each) as templates, for 25 cycles (95 °C, 10 s; 58 °C, 10 s; 68 °C, 15 s). The second round of PCRs (50 μL) were performed by using 0.4 μL of the first PCR products as templates, and combinations of nicking endonuclease-containing chimeric primer pairs Pgs-nick-# and Pps-nick-# (0.2 mM each) and universal primers Pps-R and Pgs-L with *Bsa*I sites (0.2 mM each) for NEMDA cloning. Purified PCR products of sgRNA expression cassettes (~15–20 ng each) and pYLCRISPR/Cas9Pubi-H (~100 ng) were mixed and digested by Nb.BtsI (10 units) and *Bsa*I (10 units) at 37 °C for 1 h in a total volume of 10 μL, and then incubated at 80 °C for 20 min for heat inactivation and denaturation, followed by renaturation at room temperature for 10 min, finally adding T4 DNA ligase (35 units, Takara, China) and 1.0 mM ATP at 37 °C for 30 min. The assembled constructs with multiple five sgRNA expression cassettes were directly used to transform commercial *E. coli* competent cells. The construct pYLCRISPR/Cas9Pubi-T5s was confirmed by PCR, digestion, and DNA sequencing. Primers used in CRISPR/Cas9 vector construct are listed in Supplementary Table S2.

### 3.5. Agrobacterium-Mediated Transient Expression and Stable Transformation

*Agrobacterium*-mediated transient expression was achieved in tobacco *N. benthamiana* leaves, according to previous reports with a slight modification [25,26]. Briefly, the ihpRNA plasmids, pRNAi-GUS, pRNAi-GFP, and pRNAi-NbPDS, and control plasmids pCAMBIA-1305 (35S: GUS) and pCAMBIA-1302 (35S:GFP) were transformed into *A. tumefaciens* strain GV3101, respectively. Each transformed *Agrobacterium* was cultured in YEP medium plus 50 mg/L kanamycin and 100 mg/L Rifampicin, overnight, and then centrifuged to collect the bacteria, and diluted with infiltration buffer to a final OD600 ~0.3, finally incubated at 25 °C for 2 h in the dark before agroinfiltration of *N. benthamiana* plants using a 1-mL needleless syringe. Equal volumes of the above agroinfiltration buffer were mixed and used for co-transformation. The ihpRNA plasmid pRNAi-OsPDS and the pYLCRISPR/Cas9Pubi-T5s vector were transformed into *A. tumefaciens* strain EHA105, respectively, for *Agrobacterium*-mediated rice transformation, according to our previous procedure [27].

### 3.6. RNA Isolation and qRT-PCR Analysis

Total RNA was isolated from samples (tobacco leaves, rice leaves, and calli) using Trizol reagent (Invitrogen, USA), and then treated with RNase-free DNase I (Takara, Dalian, China). First-strand cDNA was synthesized from 1 μg of DNase-treated RNA with an M-MLV reverse transcriptase kit (Promega, USA) in a total volume of 20 μL, using oligo (dT) 15 primer. All qRT-PCR assays were performed in three biological and three technical replicas on the BioRad IQ5 real-time PCR detection system. The relative expressions of interesting genes (*GFP*, *GUS,* and *NbPDS*) were calculated using the formula of the comparative Ct method 2^−ΔΔCt^ [28,29], and *NbActin* was used as an internal control to normalize gene expression. The transcript levels of the *OsPDS* gene were measured using the formula 2^−ΔCt^ [28,29], by normalizing to the expression levels of *OsActin1*. The primer sequences for RT-PCR or qRT-PCR are listed in Supplementary Table S2.

## 4. Conclusions

In summary, we have developed a simple, flexible, and low-cost NEMDA method for effective DNA fragments assembly and vector constructions. In fact, this approach can be used not only to construct the most commonly used vector tools for reverse genetics (such as RNAi and CRISPR/Cas9), but also to rapidly construct overexpression vectors for genes (by simply adding the corresponding nicking endonuclease sites on both side of the gene ORF) (Supplementary Figure S4). Due to these practicalities, we expect it to be widely utilized in DNA fragments assembly and plasmid construction for the large-scale analysis of plant functional genomics.

## Supplementary Materials

The following are available online at https://www.mdpi.com/2223-7747/9/9/1090/s1: Figure S1: Schematic diagram of pRNAi-NE vector construction; Figure S2: Zero-background cloning of intron-containing hairpin RNA (ihpRNA) constructs; Figure S3: Cloning and identification of pYLCRISPR/Cas9Pubi-T5s construct; Figure S4: Schematic diagram of NEMDA strategy for plant gene, or sense-fragment or antisense-fragment overexpression vectors construction with pRNAi-NE. Table S1: All primers used in assembly and identification of RNAi constructs; Table S2: All primers used in assembly and identification of the multiplex CRISPR/Cas9 construct.

## References

[1] Zhang Y., Yang S. (2015). Methods for Construction of Transgenic Plant Expression Vector: A Review. Chin. J. Biotechnol..

[2] Housden B.E., Perrimon N. (2016). Comparing Crispr and Rnai-Based Screening Technologies. Nat. Biotechnol..

[3] Ho S.N., Hunt H.D., Horton R.M., Pullen J.K., Pease L.R. (1989). Site-Directed Mutagenesis by Overlap Extension Using the Polymerase Chain Reaction. Gene.

[4] Bryksin A., Matsumura I. (2013). Overlap Extension Pcr Cloning. Methods Mol. Biol..

[5] Knight T. (2003). Idempotent Vector Design for Standard Assembly of Biobricks. Tech rep, MIT Synthetic Biology Working Group Technical Reports. http://hdl.handle.net/1721.1/21168.

[6] Shetty R.P., Endy D., Knight T.F. (2008). Engineering Biobrick Vectors from Biobrick Parts. J. Biol. Eng..

[7] Magnani E., Bartling L., Hake S. (2006). From Gateway to Multisite Gateway in One Recombination Event. BMC Mol. Biol..

[8] Jeong J.-Y., Yim H.-S., Ryu J.-Y., Lee H.S., Lee J.-H., Seen D.-S., Kang S.G. (2012). One-Step Sequence-and Ligation-Independent Cloning as a Rapid and Versatile Cloning Method for Functional Genomics Studies. Appl. Environ. Microbiol..

[9] Engler C., Kandzia R., Marillonnet S. (2008). A One Pot, One Step, Precision Cloning Method with High Throughput Capability. PLoS ONE.

[10] Engler C., Gruetzner R., Kandzia R., Marillonnet S. (2009). Golden Gate Shuffling: A One-Pot DNA Shuffling Method Based on Type Iis Restriction Enzymes. PLoS ONE.

[11] Gibson D.G., Young L., Chuang R.-Y., Venter J.C., Hutchison C.A., Smith H.O. (2009). Enzymatic Assembly of DNA Molecules up to Several Hundred Kilobases. Nat. Meth..

[12] Hillson N.J., Rosengarten R.D., Keasling J.D. (2012). J5 DNA Assembly Design Automation Software. ACS Synth. Biol..

[13] Zhu Q.-L., Yang Z.-F., Zhang Q.-Y., Chen L.-T., Liu Y.-G. (2014). Robust Multi-Type Plasmid Modifications Based on Isothermal in Vitro Recombination. Gene.

[14] Zheleznaya L., Kachalova G., Artyukh R., Yunusova A., Perevyazova T., Matvienko N. (2009). Nicking Endonucleases. Biochemistry (Moscow).

[15] Abrosimova L., Kisil O., Romanova E., Oretskaya T., Kubareva E. (2019). Nicking Endonucleases as Unique Tools for Biotechnology and Gene Engineering. Russ. J. Bioorg. Chem..

[16] Yang J., Zhang Z., Zhang X.A., Luo Q. (2010). A Ligation-Independent Cloning Method Using Nicking DNA Endonuclease. Biotechniques.

[17] Hu X., Liu Y.-G. (2006). Construction of RNAi Vectors and the Use for Gene Silencing in Rice. Mol. Plant Breed..

[18] Wang R.-Y., Shi Z.-Y., Guo Y.-Y., Chen J.-C., Chen G.-Q. (2013). DNA Fragments Assembly Based on Nicking Enzyme System. PLoS ONE.

[19] Yan P., Shen W., Gao X., Li X., Zhou P., Duan J. (2012). High-Throughput Construction of Intron-Containing Hairpin RNA Vectors for RNAi in Plants. PLoS ONE.

[20] Han J.-Y., Chung J., Kim J., Seo E.-Y., Kilcrease J.P., Bauchan G.R., Lim S., Hammond J., Lim H.-S. (2016). Comparison of Helper Component-Protease RNA Silencing Suppression Activity, Subcellular Localization, and Aggregation of Three Korean Isolates of Turnip Mosaic Virus. Virus Genes.

[21] Ma L., Lukasik E., Gawehns F., Takken F.L.W. (2012). The Use of Agroinfiltration for Transient Expression of Plant Resistance and Fungal Effector Proteins in *Nicotiana benthamiana* Leaves. Methods Mol. Biol..

[22] Ma X., Zhang Q., Zhu Q., Liu W., Chen Y., Qiu R., Wang B., Yang Z., Li H., Lin Y. (2015). A Robust CRISPR /Cas9 System for Convenient, High-Efficiency Multiplex Genome Editing in Monocot and Dicot Plants. Mol. Plant..

[23] Zeng D., Liu T., Ma X., Wang B., Zheng Z., Zhang Y., Xie X., Yang B., Zhao Z., Zhu Q. (2020). Quantitative Regulation of Waxy Expression by CRISPR/Cas9-Based Promoter and 5’utr-Intron Editing Improves Grain Quality in Rice. Plant Biotechnol. J..

[24] Hao Y., Zong W., Zeng D., Han J., Chen S., Tang J., Zhao Z., Li X., Ma K., Xie X. (2020). Shortened Snrna Promoters for Efficient CRISPR /Cas-Based Multiplex Genome Editing in Monocot Plants. Sci. China Life Sci..

[25] Sparkes I.A., Runions J., Kearns A., Hawes C. (2006). Rapid, Transient Expression of Fluorescent Fusion Proteins in Tobacco Plants and Generation of Stably Transformed Plants. Nat. Protoc..

[26] Xu G., Sui N., Tang Y., Xie K., Lai Y., Liu Y. (2010). One-Step, Zero-Background Ligation-Independent Cloning Intron-Containing Hairpin RNA Constructs for Rnai in Plants. New Phytol..

[27] Zhu Q., Zeng D., Yu S., Cui C., Li J., Li H., Chen J., Zhang R., Zhao X., Chen L. (2018). From Golden Rice to Astarice: Bioengineering Astaxanthin Biosynthesis in Rice Endosperm. Mol. Plant..

[28] Zhu Q., Sui S., Lei X., Yang Z., Lu K., Liu G., Liu Y.-G., Li M. (2015). Ectopic Expression of the Coleus R2r3 Myb-Type Proanthocyanidin Regulator Gene Ssmyb3 Alters the Flower Color in Transgenic Tobacco. PLoS ONE.

[29] Bustin S.A., Benes V., Garson J.A., Hellemans J., Huggett J., Kubista M., Mueller R., Nolan T., Pfaffl M.W., Shipley G.L. (2009). The MIQE Guidelines: Minimum Information for Publication of Quantitative Real-time PCR Experiments. Clin. Chem..

