# Supplementary material for: Nicking Endonuclease-Mediated Vector Construction Strategies for Plant Gene Functional Research
Source: Plants (Basel). 2020 Aug 25;9(9):1090. doi: 10.3390/plants9091090 (PMC7570347; doi:10.3390/plants9091090)
Supplement: Supplementary file 1 [file plants-09-01090-s001.pdf]

Article

Nicking Endonuclease-Mediated Vector Construction Strategies for Plant Gene Functional Research

Qi Gong [1,2,3,†], Bin Wang [1,2,3,†], Xubiao Lu [3,†], Jiantao Tan [1,3], Yuke Hou [1,3], Taoli Liu [1,3], Yao-Guang Liu [1,2,3,*] and Qinlong Zhu [1,2,3,*]

[1] State Key Laboratory for Conservation and Utilization of Subtropical Agro-Bioresources, Guangzhou 510642, China; gongqi@stu.scau.edu.cn (Q.G.); wbin313@gmail.com (B.W.); TJT@scau.edu.cn (J.T.); houyuke@stu.scau.edu.cn (Y.H.); liutaoli@stu.scau.edu.cn (T.L.)

[2] Guangdong Laboratory for Lingnan Modern Agriculture, Guangzhou 510642, China

[3] College of Life Sciences, South China Agricultural University, Guangzhou 510642, China; lxbiao648@gmail.com

* Correspondence: ygliu@scau.edu.cn (Y.-G.L.); zhuql@scau.edu.cn (Q.Z.); Tel.: +86-20-85281908 (Y.-G.L.); +86-20-85288395 (Q.Z.)

† These authors contributed equally to this paper.

Supplementary Materials:

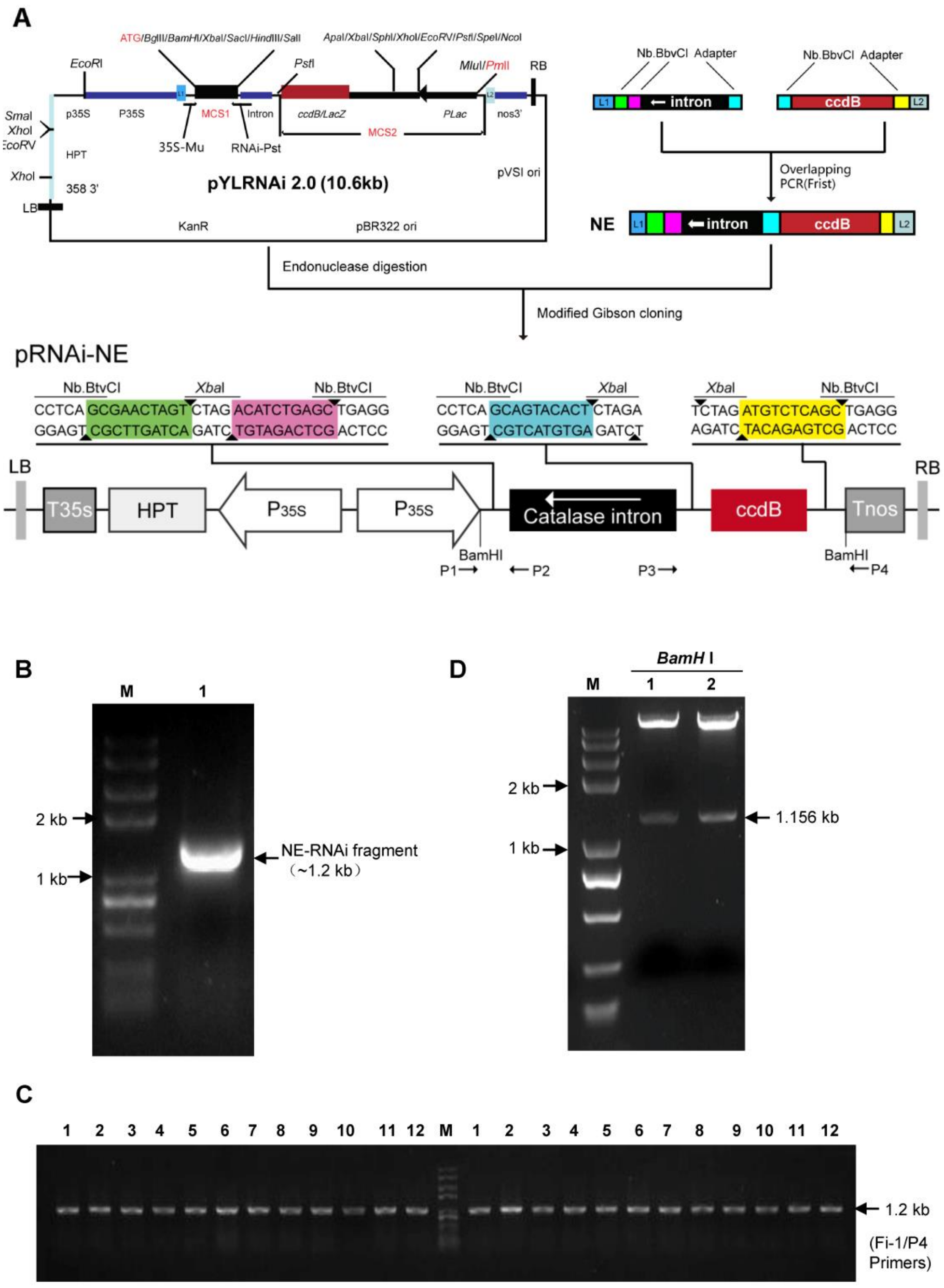

Supplemental Figure S1. Schematic diagram of pRNAi-NE vector construction. (**A**) pRNAi-NE was constructed based on pYLRNAi 2.0 vector through modified Gibson cloning [13]. (**B**) Overlappong PCR amplification of NE-RNAi fragment. (**C**) Colony PCR of pRNAi-NE using primes Fi-1 and P4 lised in Table S1. (**D**) Digestion identification of pRNAi-NE with *BamH*I.

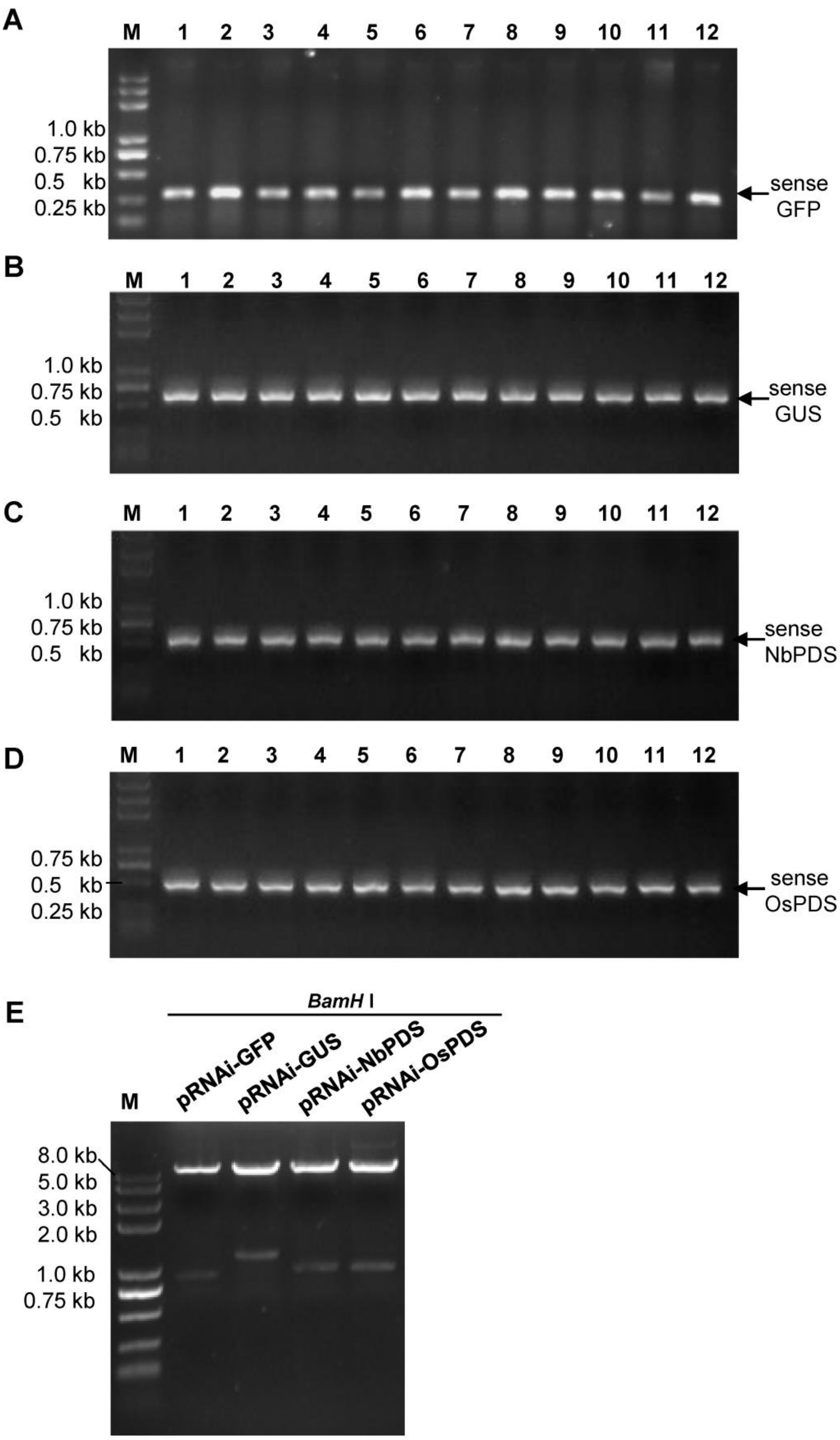

Supplemental Figure S2. Zero-background cloning of intron-containing hairpin RNA (ihpRNA) constructs. (**A**) Colony PCR identification of pRNAi-GFP by amplifying sense *GFP* fragment. (**B**) Colony PCR identification of pRNAi-GUS by amplifying sense GUS fragment. (**C**) Colony PCR identification of pRNAi-NbPDS by amplifying sense NbPDS fragment. (**D**) Colony PCR identification of pRNAi-OsPDS by amplifying sense OsPDS fragment. (**E**) BamHI-digestion identification of pRNAi-GFP, pRNAi-GUS, pRNAi-NbPDS and pRNAi-OsPDS plasmids. All primers used in clonly PCR are listed in Table S1.

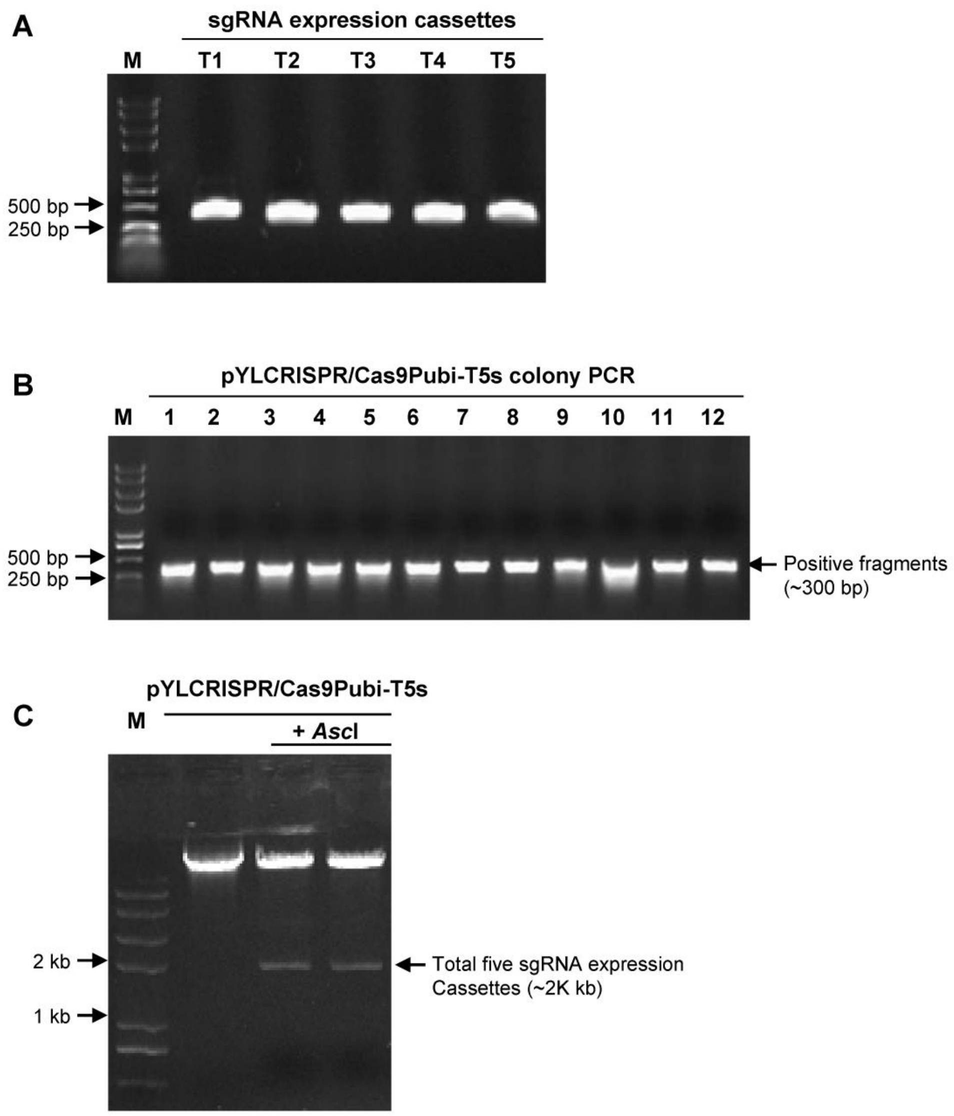

Supplemental Figure S3. Cloning and identification of pYLCRISPR/Cas9Pubi-T5s construct. (**A**) Splicing five sgRNA expression cassettes for T1-T5 targets. (**B**) Colony PCR identification of pYLCRISPR/Cas9Pubi-T5s using primers F1 and R1. (**C**) *Asc*I-digestion identification of pYLCRISPR/Cas9Pubi-T5s plasmid. All primers used in identification of CRISPR/Cas9 plasmid are listed in Table S2.

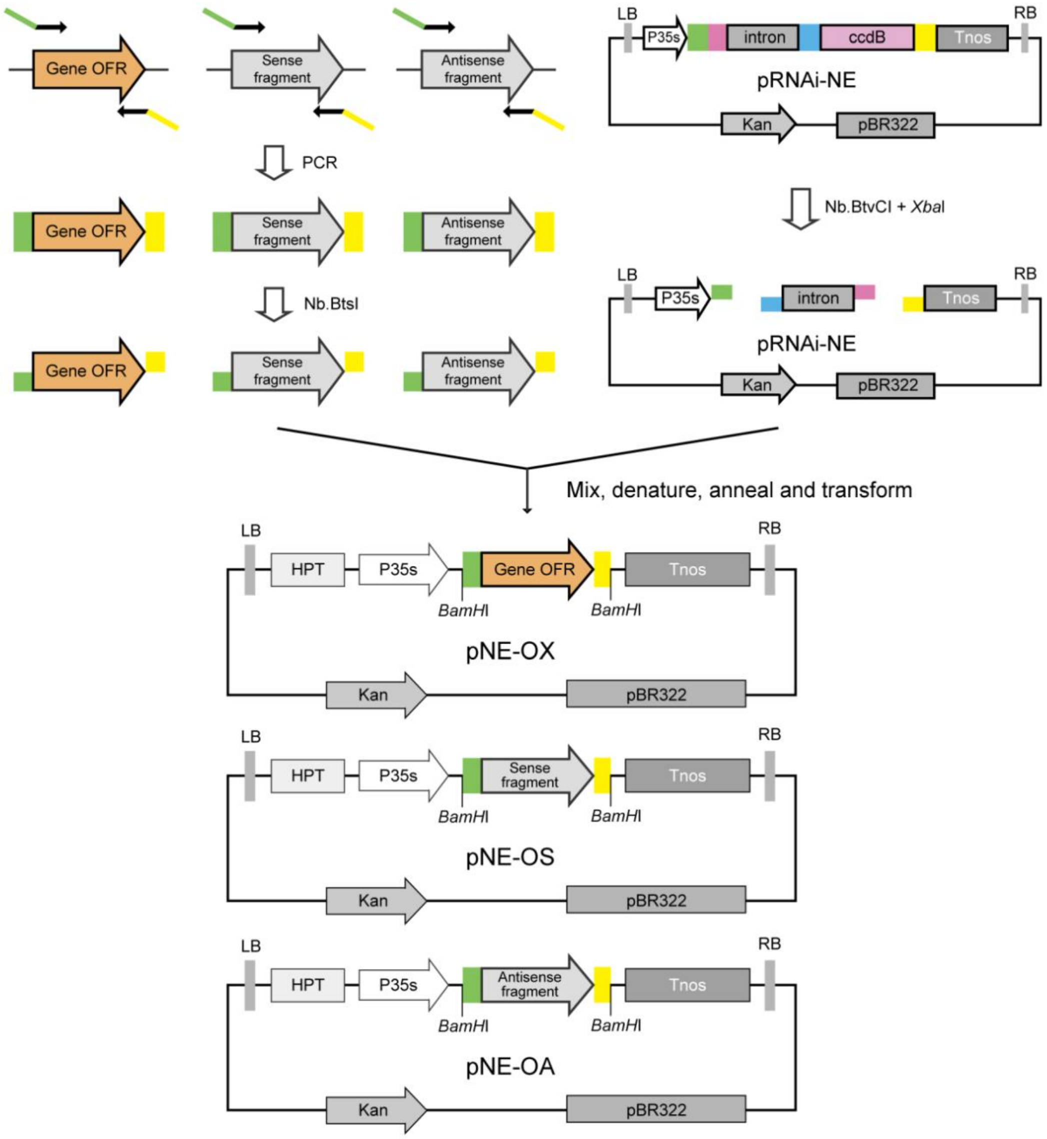

Supplemental Figure S4. Schematic diagram of NEMDA strategy for plant gene, or sense-fragment or antisense-fragment overexpression vectors construction with pRNAi-NE. Target gene open reading frames (ORF) was amplified by chimic primers with Nb.BtsI sites. The unpurified ORF PCR products digested by Nb.BtsI are mixed, in one tube, with unpurified pRNAi-NE vector digested by Nb.BtvCI and *Xba*I, then for heat inactivation of these restriction endonucleases (denaturation), anneal. The T4 DNA ligase could be used to increase construction efficiency. The reaction product was directly transformed into *E. coli* competent cells to produce the expected overexpression plasmid pNE-OX. The sense- or antisense-overexpression vectors for target genes silencing can also be obtained in a similar manner.

Supplementary Table S1. All primers used in assembly and identification of RNAi constructs.

Primer	Position	Squence（5′-3′）
Fi-1	intron-F	CCTCAGCGAACTAGTCTAGACATCTGAGCTGAGGCTGTAACTATCATCATCATCATC
Fi-2	intron-F	GACTCTTGACCATGGTAGATCTGGATCCTCAGCGAACTAGTCTAGAC
Ri	intron-R	AACTGTCTAGAGTGTACTGCTGAGGGTAAATTTCTAGTTTTTCTC
Fc	ccdB-F	CCTCAGCAGTACACTCTAGACAGTTAGCCCACGTGTTGAGAC
Rc-1	ccdB-R	GATGGATCCTCAGCTGAGACATCTAGACTGCAGACGCGTTGGCCGATTC

Rc-2	ccdB-R	CTGGTCACCAATTCACACGTGATGGATCCTCAGCTGAGACATCTAGA
P1	Check-F	GACTCTTGACCATGGTAGATC
P2	Check -R	CATAGCTTTAACTGATAATCTG
P3	Check -F	AAGAGAAAAGGGTCCTAACCAAG
P4	Check -R	ATCGGGGAAATTCGAGCTGGTC
FS-GFP	Sense-F	**GCGAACTAGT**CACTGCGGACGACGGCAACTACAAGAC
RS-GFP	Sense-R	**GCAGTACACT**CACTGCGTGCTCAGGTAGTGGTTGTC
FA-GFP	Antisense-F	**GCTCAGATGT**CACTGCGTGCTCAGGTAGTGGTTGTC
RA-GFP	Antisense-R	**GCTGAGACAT**CACTGCGGACGACGGCAACTACAAGAC
FS-GUS	Sense-F	**GCGAACTAGT**CACTGCGTCAGCGTTGAACTGCGTGATG
RS-GUS	Sense-R	**GCAGTACACT**CACTGCCGAAACCAATGCCTAAAGAGAG
FA-GUS	Antisense-F	**GCTCAGATGT**CACTGCCGAAACCAATGCCTAAAGAGAG
RA-GUS	Antisense-R	**GCTGAGACAT**CACTGCGTCAGCGTTGAACTGCGTGATG
FS- NbPDS	Sense-F	**GCGAACTAGT**CACTGCGAAGCTAGTGGGAGTTCCTGTG
RS- NbPDS	Sense-R	**GCAGTACACT**CACTGCGCAAGGGCCGACAGGGTTCAC
FA- NbPDS	Antisense-F	**GCTCAGATGT**CACTGCCGAAACCAATGCCTAAAGAGAG
RA- NbPDS	Antisense-R	**GCTGAGACAT**CACTGCGCAAGGGCCGACAGGGTTCAC
FS- OsPDS	Sense-F	**GCGAACTAGT**CACTGCTCTTGAAGCTTCTTGTACCTCAAG
RS- OsPDS	Sense-R	**GCAGTACACT**CACTGCTTCAGAATCTTTGCTTTACTC
FA- OsPDS	Antisense-F	**GCTCAGATGT**CACTGCTTCAGAATCTTTGCTTTACTC
RA- OsPDS	Antisense-R	**GCTGAGACAT**CACTGCTCTTGAAGCTTCTTGTACCTCAAG
FGFP	RT-PCT, qRT-PCR	TAAACGGCCACAAGTTCAGC
RGFP		GTAGGTCAGGGTGGTCACGA
FGUS	RT-PCT, qRT-PCR	AATCCATCGCAGCGTAATGC
RGUS		ACCACCTGCCAGTCAACAGA
FNbPDS	RT-PCT, qRT-PCR	CCCGAAGATTGACAAAGGAC
RNbPDS		CGTGAGGAAGTACGAAACGA
FNbActin	RT-PCT, qRT-P	CGTAGCCCTAGACTATGAGCAGG
RNbActin		GCACTATGTTTCCGTAGAGGTCC
FOsPDS	RT-PCT, qRT-PC	TGCCAAACAAGCCAGGAGAA
ROsPDS		CCATTGCTGGCAAAAGTCCA
FOsActin	RT-PCT, qRT-PCR	GCATCTCTCAGCACATTCCA
ROsActin		ACCACAGGTAGCAATAGGTA

Note: The underlined bases are the recognition site of NB.btsI; The black bold base represents the single-chain complementary overhanding end sequence produced by NB.btsI.

Supplementary Table S2. All primers used in assembly and identification of the multiplex CRISPR/Cas9 construct.

Primer	Position	Squence（5′-3′）
U-F	Promoter-F	CTCCGTTTTACCTGTGGAATCG
gRNA-R	SgRNA-R	CGGAGGAAAATTCCATCCAC
OsU6aT1-R BHY	U6a(T1)-BHY	**GGTACGTGCGCCGCTCCGAC**GGCAGCCAAGCCAGCA
gRT1-F	SgRNA(T1)-BHY	**TCGGAGCGGCGCACGTACC**GTTTCAGAGCTAGAAAT
OsU6bT2-R	U6b(T2)-BHY	**CCACCGACAACGCGAACGTGC**AACACAAGCGGCAGC
gRT2-F	SgRNA(T2)-BHY	**CACGTTCGCGTTGTCGGTGG**GTTTCAGAGCTAGAAAT
OsU6cT3-R	U6c(T3)-EHY	**ATAGCGCGAAGGTGCCGAAC**TGAGCCTCAGCGCAG
gRT3-F	SgRNA(T3)-EHY	**TTCGGCACCTTCGCGCTAT**GTTTCAGAGCTAGAAAT
OsU3T4-R	U6c(T4)-Wx	**GTCTTGCTCCAGGGGGCCAT**GCCACGGATCATCTGC
gRT4-F	SgRNA(T4)-Wx	**TGGCCCCCTGGAGCAAGAC**GTTTCAGAGCTAGAAAT

OsU3T5-R	U6c(T5)-Wx	**CAGCCACAACGCTGGTATCCT**GCCACGGATCATCTGC
gRT5-F	SgRNA(T5)-Wx	**GGATACCAGCGTTGTGGCTG**GTTTCAGAGCTAGAAAT
Pps-R	T1 cassette-F	TTCAGAggtctcTaccgACTAGTATGGAATCGGCAGCAAAGG
Pgs-nick-2	T1 cassette-R	**GCGAACTAGT**CACTGCTCCATCCACTCCAAGCTC
Pps-nick-2	T2 cassette- F	**ACTAGTTCGC**CACTGCGGCCCAATCGGCAGCAAAGGA
Pgs-nick-3	T2 cassette-R	**AGTGTACTGC**CACTGCTCCATCCACTCCAAGCTC
Pps-nick-3	T3 cassette-F	**GCAGTACACT**CACTGCGGCCCAATCGGCAGCAAAGGA
Pgs-nick-4	T3 cassette-R	**GCTCAGATGT**CACTGCTCCATCCACTCCAAGCTC
Pps-nick-4	T4 cassette-F	**ACATCTGAGC**CACTGCGGCCCAATCGGCAGCAAAGGA
Pgs-nick-5	T4 cassette-R	**ATGTCTCAGC**CACTGCTCCATCCACTCCAAGCTC
Pps-nick-5	T5 cassette-F	**GCTGAGACAT**CACTGCGGCCCAATCGGCAGCAAAGGA
Pgs-L	T5 cassette-R	AGCGTGggtctcGctcgACGCGTATCCATCCACTCCAAGCTC
F1	Check-F	TCGGAGCGGCGCACGTACCgtttcagagctagaaat
R1	Check-R	CCACCGACAACGCGAACGTGCaacacaagcggcagc

Note: Bold and underlined indicate editing sites; The underlined bases are the recognition site of NB.btsI; The black bold base represents the single-chain complementary overhanging end sequence produced by NB.btsI digestion; Boxed bases indicate *Bsa*I sites.
